# Supplementary material for: Interventions for the Prevention of Oral Mucositis in Patients Receiving Cancer Treatment: Evidence from Randomised Controlled Trials
Source: Curr Oncol. 2023 Jan 10;30(1):967–80. doi: 10.3390/curroncol30010074 (PMC9858113; doi:10.3390/curroncol30010074)
Supplement: Supplementary file 1 [file curroncol-30-00074-s001.zip › curroncol-2112768-SI.pdf]

Supplementary tables

Table S1. Summary finding of article reporting preventative interventions for radiotherapy-induced OM

Ref.	Author, Year	P	I	C	O	Summary of Findings
	Author, Year	Patient Population and Size	Intervention	Control/Comparison	Outcomes	Summary of Findings
7	Epstein, J. B., Stevenson-Moore, P., Jackson, S., et al. (1989)	43 Patient scheduled for radiation therapy to oropharyngeal region, 18 or more years old.	Drug rinse consisted of 1.5mg/mL Benzydamine hydrochloride (Bzd)	Placebo rinse	Severity of OM and pain recorded weekly using visual analogue scales (VAS). Amount of analgesic usage.	The total mucositis score was less in the Bzd group than the placebo group (p = 0.00 1). The average area of mucositis during radiation therapy and the maximum mucositis score was significantly less in the Bzd versus the placebo groups (p = 0.05). The maximum size of ulceration and the total area of ulceration was significantly less in the Bzd group (p = 0.04; p = 0.05). Although not statistically significant, less pain reported in Bzd group (p=0.08), less pain with eating (0=0.09), pain reduction reported with Bzd (p=0.067) and reports of anaesthesia in the Bzd group (p=0.10).
8	Sheibani, K. M., Mafi, A. R., Moghaddam, S., et al. (2015).	51 patients with head and neck carcinoma receiving external RT treatment dose of at lease 5000cGY with at least two OM sites included in the planned RT treatment; 26 treatment (Age 53.2 ± 11.2), 25 placebo (Age 52.1 ± 12.0)	0.15% Benzydamine Oral Rinse	Placebo - same appearance and taste consisting of only the vehicle	Primary outcome was to assess the efficacy of intervention in reducing the signs and symptoms of OM	Study showed that benzydamine hydrochloride can be helpful in reducing the degree of oral mucositis in patients undergoing RT. Initially, up to the third week, both groups did not show any significant difference in severity but by the end of week 4, the mean scores of the placebo group was statistically more than treatment group (p = 0.01) and continued until the end of treatment
11	Saarilahti, K., Kajanti, M., Joensuu, T., et al. (2002).	40 patients (20 control, 20 intervention) scheduled to receive postoperative radiotherapy for head-and-neck cnacer to a total dose of greater than/equal to 50Gy to the oral and oropharyngeal mucosa; 21 GM-CSF (Age 43-87); 19	Comparison of Granulocyte-Macrophage Colony-Stimulating Factor (GM-CSF) and Sucralfate Mouthwashes	Granulocyte-Macrophage Colony-Stimulating Factor vs. Sucralfate Mouthwashes	Primary outcome was identifying the presence and severity of OM.<br><br>Secondary Outcome was looking at mucosal pain, body weight, serum prealbumin, total white blood cell and neutrophil counts, Tolerability of GM-CSF and sucralfate mouthwashes, and RT interruptions	Overall, the mucositis scores tended to be less severe in the GM-CSF-group (p = 0.072), and the difference between the curves tended to increase during the radiation course, reaching a maximum at Week 6. Healing of oral mucosal membranes despite continued radiation took place in 5 (24%) of the 21 patients assigned to the GM-CSF group; however,

		sucralfate (Age 24-72); treated as outpatients				none of the 19 patients randomized to sucralfate mouthwashes showed a similar effect (p = 0.049). OM pain reported was on average milder in the GM-CSF group (p = 0.058), and the difference between the groups tended to increase toward the end of RT.
22	Ameri, A., Poshtmahi, S., Heydarirad, G., et al. (2021).	64 patients with HNC received external beam radiotherapy for 5 days per week.	honey-lemon spray	benzydamine hydrochloride spray	World Health Organization (WHO) mucositis grading scale	The physician's inspection did not show a significant difference in the frequency and mucositis score of OM (p = 0.051) during the 5-week treatment in both groups. Regarding the grade of OM occurring in patients during the study, 10 patients had grade 3 mucositis in the intervention group against 14 in the control group (p = 0.292). There were no significant group differences in mucositis severity between patients treated with honey-lemon spray and benzydamine hydrochloride.
16	Delavarian, Z., Pakfetrat, A., Ghazi, A., et al. (2019)	32 HNC patients undergoing radiotherapy, 18 or older.	nanoCurcumin orally	placebo	Occurrence and severity of OM is graded based on NCI CTC version 2 scale.	The grade of OM was significantly lower in study group compared to control group over the 6 weeks (p<0.05). Nanomicelle curcumin is an effective agent in the prevention of OM or reducing its severity.
17	Demir Doğan, M., Can, G., & Meral, R. (2017)	80 HNC scheduled to undergo RT to the oropharyngeal mucosa, 18 and above, no metastasis.	Black mulberry molasses	No black mulberry molasses	NCI-CTCAE used to grade OM, Oral assessment guide used to assess the clinical and functional changes in oral cavity, UW-QOL to ggrade QoL.	The incidence of OM was significantly higher in control group than experiomental group at week 3, 4, 7 (chi square test, p<0.05). The severity of OM was significantly higher in control group at week 4 and 6 (chi square test, p<0.05). The average pain score was lower in experimental group at weekly follow-ups. Pain develop earlier in control group (chi square, p<0.05). The QOL is signifiantly lower in control group (t=3.76, p<0.00). Black mulberry molasses usage is an effective intervention in the prevention of the radiation-induced mucositis of head and neck cancer patients.
18	Elyasi, S., Hosseini, S., Niazi Moghadam, M. R., et al. (2016)	27 HNC patients scheduled for radiotherapy for the first time, aged 18-65	Oral silymarin tablets	Placebo tablets	Severity of OM measured by WHO grading scale and NCI-CTC scale.	The median WHO and NCI-CTC scores were significantly lower in silymarin group at the end of fourth to sixth week (p<0.05). The scores increased significantly in both groups during radiotherapy (p,0.001), but there was a delay for mucositis development and progression in silymarin group.

						Prophylactic administration of conventional form of silymarin tablets could significantly reduce the severity of radiotherapy induced mucositis and delay its occurrence in patients with head and neck cancer.
23	Hosseini, S., Rezaei, S., Moghaddam, M. R. N., et al. (2021).	31 Patients with head and neck cancer who underwent radiotherapy for the first time, age range between 18–65 years, total radiation dose 50–70 Gy.	Nano-silymarin solution	Placebo solution	Patients were assessed according to the European Organization for Research and Treatment of Cancer Scale (EORTC) for the prevention of OM.	The median EORTC scores were not significantly different between silymarin and placebo groups at the end of the sixth week ($p > 0.05$). According to the results of this study, the nano-solution of silymarin could not prevent mucositis in patients with head and neck cancer undergoing radiation therapy in comparison with placebo.
19	Putwatana, P., Sanmanowong, P., Oonprasertpong, L. et al (2009)	Patients 18 years and older, diagnosed with head and/or neck cancer, planning to receive radiation alone or in combination with other treatment, with no preexisting oral disease, with no concomitant drug use (such as other mouth rinse solutions, antibiotics, oral antifungals, and antivirals)<br><br>60 (30 control, 30 intervention)	Glycerin payayor, a Clinacanthus nutans extract.<br>2 drops of glycerin payayor 3 to 5 times a day	Benzydamine hydrochloride (Difflam, 3M, Australia)<br>Dosage was 15 mL Difflam for mouth rinsing 3 times a day	The World Health Organization (WHO) Mucositis Grading System was used to evaluate the severity of oral mucositis	The mean (SD) time to the onset of oral mucositis in the payayor group was 2.55 (1.24) weeks, whereas in the BZD group, it was 1.02 (0.44) weeks. It was significantly different ($t = 2.88$, $P < .001$). The duration of oral mucositis, pain, xerostomia, and taste alteration between the 2 groups was at least 1 week apart, showing a significant difference ($P < .01$)<br><br>Mean severity scores were significantly different between the payayor group (0.20-0.95) and the BZD group (0.96-1.95) ($P < .001$).
20	Rezaeipour, N., Jafari, F., Rezaeizadeh, H. et al(2017).	23 adult patients with H and N cancers, who came for radiotherapy in Iran	herbal compound (containing A.digitata and M.sylvestris) 3 times per day for 7 weeks from beginning of radiotherapy to 2 weeks later	placebo sachets	The efficacy of treatment on mouth pain score (MPS) was assessed by using visual analog scale (VAS) and mucositis grade was evaluated by investigator according to WHO scale in every visit.	Drug effect, time effect, and time-drug interaction on average mucositis score are statistically significant ($p < 0.0001$)<br><br>The average of mucositis severity showed significant difference between 2 groups so that mucositis score in control group was higher than experimental group in every weekly cutting ($p < 0.0001$)<br><br>Mouth pain score is also significantly lower in the experiemental group in each time period ($p < 0.001$)
21	Wu, M. H., Yuan, B., Liu, Q. F. et al. (2007).	60 patients (age 34-62) that conform to the diagnostic standard of head-neck	Qingre Liyan Decoction	Dobell's Solution	Primary Outcome - Study effect of Qingre Liyan Decoction in the prevention and treatment of acute radiative OM	The incidence of Acute Radiative Oral Mucositis (AROM) is lower and the effect in preventing AROM is higher in the trial

		carcinoma and be suitable for RT, with no severe functional abnormality in the heart, liver and kidney and no remote metastasis of carcinoma. (30 intervention, 30 control)			severity of AROM was graded into 5: Grade 0: no change; Grade I: hyperaemia with mild pain but no need of analgesic; Grade II: flaky mucositis bloody secretion, moderate pain and need of analgesic; Grade III: fused fibrinous mucositis with severe pain and need of narcotics; Grade IV: ulcerative mucositis with bleeding, and necrosis.<br><br>Secondary Outcome - Explore mechanism of QRLYD by detecting Epidermal Growth Factor and T Lymphocytes (CD3, CD4 and CD8)	group than those in the control group (P < 0.05). The EGF in saliva, and CD4 and CD8 in the blood of patients in the trial group were higher than those in the control group (P < 0.05)
10	Dodd, M. J., Cho, M. H., Cooper, B. A.,et al. (2022)	91 patients with HNC and scheduled to receive a RT, aged 18 and above	Granulocyte macrophage colony stimulating factor (GM-CSF) mouthwash	Salt and soda mouthwash	Occurrence and grades on OM according to RTOG-ARMSC scale, functional status, pain, chewing and swallowing during and after RT.	No significant differences were found in the occurrence (exact logistic regression, p<0.09) and grades (ordinal logistic regression p=0.95) of OM in the patients who used GM-CSF vs. Salt and Soda in the prevention phase. Patients' ratings of functional status, pain severity, chewing ability, and swallowing difficulty were not significantly different among the groups (multilevel regression).
12	Arora, H., Pai, K. M., Maiya, A., Vidyasagar, M. S., et al. (2008).	24 patients with oral cancer (55 to 59 yo)	Helium-Neon (He-Ne) LASER (wavelength 632.8 nm and output of 10 mW, daily before radiotherapy	control	Severity of oral pain using the Numeric Rating Scale (NRS). Need for supplemental analgesic medication (World Health Organization [WHO] analgesic ladder). Severity of dysphagia was assessed daily using Functional Impairment Scale (FIS). Severity of mucositis was assessed using the Radiation Therapy Oncology Group/European Organisation for Research & Treatment of Cancer (RTOG/EORTC) scoring system.	pain progressively decreased in laser group as compared to the control; P = .033. support the contention that laser therapy applied prophylactically during radiotherapy can reduce the severity of mucositis, severity of pain, and functional impairment.
13	Arun Maiya, G., Sagar, M. S., & Fernandes, D. (2006).	50 patients with carcinoma of oral cavity with stages II-IV a being uniformly treated with curative total tumour dose of 66 Gy in 33 fractions over 6 wk .	He-Ne laser (wavelength 632.8 nm and output of 10 mW	oral analgesics and local application of anaesthetics, 0.9 per cent saline	WHO scale for mucositis2 and visual analogue scale for pain evaluation3	Significant difference in pain and mucositis (P<0.001) between the two groups. At the end of radiotherapy (after 6 wk) mean pain sure and mucositis grade were significantly lower (P<0.001) in the study group compared to control. The low-level He-Ne laser therapy during the radiotherapy treatment was found to be effective in preventing and treating the mucositis in head and neck cancer patients.
14	Bensadoun, R. J., Franquin, J. C.,	30 patients with carcinoma of the oropharynx,	Low-energy He-Ne laser (LEL),	sham-treatment	The criteria used for evaluation were the standard WHO staging for mucositis (Table	The mean grade of mucositis during radiotherapy was 2.1B0.26 for the group

	Ciais, G., et al. (1999).	hypopharynx and oral cavity, treated by radiotherapy alone			2) and a modified visual analogue scale for pain (patient self-evaluation).	without laser (L–) and 1.7B0.26 for the group with laser (L+) (P=0.01). Preventive use of laser application significantly (P=0.025) reduced oral pain for the treatment area, as assessed by patients, over a 7-week period. Low-energy He/Ne laser (LEL) seems to be a safe and efficient method for the prevention of radiation-induced stomatitis, and reduces oral pain
15	Kauark-Fontes, E., Migliorati, C. A., Epstein, J. B., et al. (2022).	55 patients diagnosed with OOPSCC in stage III or IV (International Union Against Cancer, 8th edition) [9], over the age of 18 years, treated with curative RT protocols as a single modality or in association with CT	Prophylactic extraoral photobiomodulation	LED sham sessions with an inactivated extraoral probe	Patients were evaluated weekly for the presence, topography, and severity of OM following the Common Terminology Criteria for Adverse Events (NCI, version 4.0, 2010), graded 0–4	The frst occurrence of OM was observed at week 1, for the placebo group (p=0.014). Later, OM onset and severity was observed for the PBM group, with frst occurrence at week 2 (p=0.009). No diference in severe OM incidence was observed (p>0.05). Prophylactic extraoral PBM can delay OM onset, reduce pain, and reduce analgesic and anti-infammatory prescription requirements.
27	Cengiz, M., Özyar, E., Öztürk, D., et al. (1999).	28 patients with head and neck cancer	sucralfate (6 g sucralfate suspension as mouth wash)	placebo	Radiation Therapy Oncology Group (RTOG) acute radiation morbidity scoring criteria for mucositis. Symptoms evaluated during radiotherapy were steady oral pain, pain during feeding, dry mouth, alteration of taste perception, tolerable consistency of food, and constipation.	There was a significant difference in radiation-induced visible mucosal changes between sucralfate and placebo groups ($p < 0.05$). Analysis of pain detected a statistically significant difference between groups. Sucralfate group experienced considerably less pain compared with the control group during feeding ($p = 0.0072$). Sucralfate significantly decreased the degree of mucositis and oral pain during feeding.
28	Abbasi Nazari, M., Sadrolhefazi, B., Nikoofar, A., et al. (2007).	24 patients with oral, nasopharynx or hypopharynx cancer who had undergone radiotherapy (daily dose 180-200 cG and total dose 6000-6500 cG)	Allopurinol mouthwash	placebo mouthwash	WHO grading system	no significant differences between groups in severity of mucositis in the first and second week (p =0.227, p=0.121 respectively). significant difference between two groups in severity of mucositis in the third, forth, fifth and sixth week of treatment (p <0.05 in each week separately). Result of this study support the hypothesis that an allopurinol mouthwash may prevents or alleviate oral mucositis induced by radiotherapy.
29	Bai, X. H., Chen, Z. M., Ma, L. H., et al. (2019).	68 patients with HNC (including nasopharyngeal cancer)	distilled water atomization inhalation at a low temperature (temperature between 4°C and 8°C)	distilled water atomization inhalation at room temperature (the distilled water was between 18°C and 24°C).	severity of OM was classified to five grades via Common Terminology Criteria for Adverse Events (CTCAE) version 4.03 combining with the Radiation Therapy Oncology Group (RTOG) criteria. Dryness	There was a significant difference in the incidence of OM between the two groups when the total radiation dose reached 30 Gy (42.4% in the intervention group vs 68.6% in the control group, P<0.05), as well

					of the oral cavity was evaluated via RTOG acute radiation morbidity scoring criteria to the salivary gland	as when the radiotherapy was completed (78.8% in the intervention group vs 91.4% in the control group, P<0.05). There were fewer patients with severe OM in the intervention group compared to the control group both at the 30 Gy radiation dose and at the completion of radiotherapy (6.0% and 18.2% in the intervention group vs 22.9% and 51.4% in the control group respectively, P<0.05). the onset time of OM in the intervention group was delayed about 4 days compared to that in the control group (16.55 days in the intervention group vs 12.63 days in the control group, P<0.05). Low-temperature atomization inhalation can reduce the incidence and severity of OM, and slow down the progression process of it.
30	de Sanctis, V., Belgioia, L., Cante, D., et al. (2019)	75 patients histologically diagnosed of head and neck carcinoma, aged 18 and above, scheduled to be treated with intensity modulated radiation therapy.	Lactobacillus brevis CD2 lozenges	sodium bicarbonate mouthwashes	OM grade assessed weekly according to NCI CTCAE version 4.0, as well as the Incidence of grade 3 or 4 oropharyngeal mucositis.<br>QoL was assessed using the FACT H&N quality of life questionnaire.<br>Pain and dysphagia were weekly recorded acording to Radiation Therapy Oncology Group Common Toxicity Criteria.	There was no statistical difference in the incidence of grade 3-4 oropharyngeal mucositis between the intervention and control groups (40.6% vs. 41.6% respectively, p=0.974). The incidence of pain, dysphagia, body weight loss and quality of life were not different between the experimental and standard arm.
119	Elsabagh, H. H., Moussa, E., Mahmoud, S. A., et al. (2020)	40 HNC patients who were scheduled for radiotherapy , 25 years old or more.	Melatonin	no melatonin	OM severity (WHO grading system) and pain NRS score at 3 and 6 weeks	92.5% of all patients have experienced oral mucositis with more severity reported in the control group (30%) compared with the test group (5%). Mean pain scores was significantly lower in test group rather than the controls after 6 weeks (p<0.001). The administration of melatonin with conventional treatment has reduced severe oral mucositis development and aided in decreasing pain.
26	Emami, H., Jalilian, M., Parvizi, A., et al. (2008)	52 HNC patients who received RT	Sucralfate mouthwash	placebo mouthwash	Severity of OM measured by WHO grading scale. Relative frequency of grade 3 OM. The time period between the beginning of radiotherapy and the appearance of OM.	Mann-Whitney U test showed that within 4 weeks of evaluation, severities of mucositis in experimental group was significantly less than those in control group, with P values of 0.02, 0.02, 0.001 and 0.004, respectively. Two-sample Kolmogorov-Smirnov test showed that the relative frequency of grade 3 mucositis in experimental group was significantly less than that in control group (100% in control group vs 57.7%) in

						experimental group (P-value = 0.0001). The time period between the beginning of radiotherapy and the appearance of mucositis was not statistically different in the two groups (P = 0.9).
24	Kiprian, D., Jarzabski, A., & Kawecki, A. (2016).	100 patients with head and neck cancer referred for radiotherapy or radiochemotherapy with radical intent	Caphosol	Standard treatment	Mucositis, xerostomia, and dysphagia were scored by radiotherapists. Subjective evaluation was made by patients.	A statistically significant difference in mean severity of early irradiation-induced side effects between the studied groups was observed with respect to: mucositis in the clinical target volume (CTV) area, mucositis in the increased dose (boost) area, dysphagia and xerostomia ($p < 0.001$ for all reactions).
25	Laali, E., Manifar, S., Kazemian, A. et al (2020).	71 (34 control, 37 prevention), Patient Selection<br>Patients with head and neck cancer who were due to receive radiation on at least two-thirds of the oral cavity were included in the study. Patients aged 18–85 years with a<br>normal renal function (creatinine clearance > 60 ml/min) and an acceptable performance status (Karnofsky performance status > 70%) without any history of RT.	Selenium 200 mg taken twice daily from first day of radiation to the end.	placebo	The World Health Organization (WHO) oral toxicity scale was used to evaluate OM	The cumulative incidence of OM (grade 1–4) was not significantly different between the two groups (97.3% in selenium and 100% in the placebo group, p value: 0.79)<br>The mean duration of OM (grade 1–4) was not different between the two groups (46.97 ± 20.26 days in the selenium and 50.44 ± 17.56 days in the placebo group, p value 0.27)<br>However, based on the selenium level before radiation, developing severe OM was statistically significant postponed in patients who had selenium levels ≥ 65 mcg/L (p value 0.04).

Table S2. Summary finding of article reporting preventative interventions for chemotherapy-induced OM

Ref NO.	Author, Year	Patient Population and Size	Intervention	Control/Comparison	Outcomes	Summary of Findings
31	Cheng, K. K. F., Molassiotis, A., & Chang, A. M. (2002).	14 patients (a) diagnosis of leukaemia, lymphoma, or paediatric solid tumor, (b) undergoing high-dose or combination chemotherapy, (c) age between 6 and 17 years of age,	preventive oral care protocol consisting of tooth brushing, 0.9% sodium chloride solu- tion and 0.2% chlorhexidine mouth rinse	did not receive the oral care protocol intervention	The Eilers' Oral Assessment Guide was used (Eilers et al. 1988), after minor modification, to measure the severity of oral mucositis.	the difference in incidence between the two groups was not statistically significant (w2=2.8, P=0.09). There were statistically significant differences in the mean oral mucositis score between the control and experimental groups across all time point evaluations (F=19.3, P=0.001). There were statistically significant differences in the mean pain score between control and experimental group across all time point evaluations (F=10.92, P=0.006). The results in this pilot study provide initial support that the oral care protocol intervention offers some promise in reducing oral mucositis for paediatric cancer patients undergoing chemotherapy.
32	Cheng, K. K. F., Molassiotis, A., Chang, A. M., et al. (2001).	42 Children between the ages of 6 to 17 years who had received high-dose or combination chemotherapy for haematological malignancies or solid tumours.	oral care protocol intervention: regular tooth brushing, rinse with 0.2% chlorhexidine mouth rinse and 0.9% saline rinse.	No oral care protocol interventio.	Incidence of oral mucositis based on the clinical manefestation of ulcerative lesions in the oral mucosa. The severity was measured by Eilers' Oral Assessment Guide after minor modification. Pain intensity was scored by Faces Scale.	A 38% reduction in the incidence of ulcerative mucositis (chi square =6.1, p=0.01). The severity of oral mucositis/mean OM score across all time points significantly reduced with the intervention (F=30.79, P=0.000002). The related pain (F=19.22, P=0.0001) were significantly reduced with the intervention. The intensity of pain was signifiantly correlated with score of OM in two groups (r=0.89, p=0.007).
33	Dodd, M. J., Larson, P. J., Dibble, S. L., et al. (1996)	222 patients who were starting a cycle of chemotherapy, over 18 years old.	Chlorhexidine mouthwash 20mL, swish for 20 seconds	Placebo mouthwash (water)	Incidence, days to onset, severity of OM using Oral Assessment Guide (OAG).	No significant difference in the incidence of OM between 2 groups (Chi square test, p=0.76). No significant difference in the days to onset of OM (p=0.40). No

						significant difference in the severity of OM (p=0.75).
34	Epstein, J. B., Vickars, L., Spinelli, J., & Reece, D. (1992)	86 patients scheduled for treatment with intensive chemotherapy, >18 yeas old.	1. chlorhexidine rinse<br>2. nystatin suspension<br>3. A combination of nystatin and chlorhexidine rinses	4. saline solution rinse (15mL/rinse)	Oral mucositis was assessed in each region of the oral cavity, and erythema and ulceration were recorded. An overall mucositis score was developed for each visit by combining the score of each area involved, dividing the total area of mucositis by the number of surfaces involved, and multiplying the result by the severity of the inflammation. Bacterial, fungal and viral cultures were obtained.	No significant differences were noted between rinse groups in measures of oral ulceration and oral mucositis (ulceration and erythema together).
37	Parkhideh, S., Zeraatkar, M., Moradi, O. et al (2022)	Patients aged 15 years and older, being a nonsmoker, able to gargle mouthwash solution, with no history of liver disease and capability of reading and communicating with staff, and able to sign the informed consents were included to the study.<br><br>70 (35 control, 35 intervention)	300mg of azithromycin suspension twice daily	Placebo mouthwash	patients were assessed for OM episode incidence as the primary outcome.<br><br>The secondary outcomes were measured in both groups as the time of mucositis occurrence, duration of OM episodes, maximum grade and, average daily degree of mucositis, the necessity to receive other mucositis therapies, need to receive and duration of PN, antibiotics utilization, length of hospital stay, days with fever, first episode of infection, and engraftment time. Also, patients were assessed for severity of the symptoms and pain, dryness of oral cavity, dysphagia and alteration taste perception, serum glutamate-pyruvate transaminase (SGPT), and serum glutamic-oxaloacetic transaminase (SGOT) (due to azithromycin side effect), Serum creatinine<br><br>National Cancer Institute Common Toxicity Criteria (NCI-CTC) version 4 mucositis scale	Developing OM significantly decreased in the treatment group (P = 0.015). Azithromycin was associated with delayed onset (7.5 ± 1.4 vs 5.3 ± 2.2, P = 0.015) and reduced duration (p = 0.045).<br><br>But there was no significant difference in the maximum grade of mucositis (p = 0.157) and average daily grade of mucositis (p = 0.298).<br><br>In terms of OM symptoms, a significant decrease was detected in the level of pain (p = 0.01), dryness of oral cavity (p < 0.001), sense of taste (p < 0.001), and dysphagia (p < 0.001) in the azithromycin suspension group.

					was used to measure the OM severity	
35	Sorensen, J. B., Skovsgaard, T., Bork, E., et al. (2008).	225 patients (Age 28-84) with previously untreated GI cancer (gastric or colorectal) receiving bolus 5-FU/leu- covorin chemotherapy; randomised into 3 groups (Chlorhexidine, Saline (Placebo), Cryotherapy); 206 patients responded to the questionnaire	Prophylactic Chlorhexidine Mouthwash (Arm A)	Placebo - saline rinse (Arm B); Cryotherapy - Crushed Ice during Chemotherapy (Arm C)	Primary outcome was to measure the severity and duration of oral mucositis	Frequency of OM grades 3 or 4 were significantly lower in Chlorhexidine ($P < 0.01$) and Cryotherapy ($P < 0.05$) arms in comparison to placebo. Duration was significantly shorter in Chlorhexidine ($P = 0.035$)and Cryotherapy ($P = 0.003$) when compared to placebo. No signifcant difference between chlorhexidine and cryotherapy.
38	Vokurka, S., Bystřická, E., Koza, V., et al. (2005).	132 patients (Age 20-72), treated with high-dose BEAM or HD-L-PAM chemotherapy followed by APBSCT. They had a healthy oral mucosa, did not require the intervention of a stomatologist. he BEAM conditioning consisted of BiCNU (carmustine) 300 mg/m2 i.v. once daily on day −6, etoposide 100 mg/m2 i.v. twice daily on days −5 to −2, Ara-C (cytarabine) 200 mg/m2 i.v. twice daily on days −5 to −2, melphalan 140 mg/m2 i.v. once daily on day −1. The HD-L-PAM conditioning consisted of melphalan 200 mg/m2 i.v. on day −1. The patients were given systemic antimycotics and antibacterial prophylaxis (fluconazole or itraconazole and norfloxacin or ciprofloxacin) and G-CSF post-transplant to support engraftment. (67 intervention, 65 control)	Povidone-Iodine Mouthwash	Saline Mouthwash	OM was assessed once daily using WHO grading on a scale of 0-4 (0 absent; 1 slight pain, erythema; 2 sore defects, can eat solids; 3 very sore defects, requires liquid diet only; 4 alimentation not possible). Oral pain was evaluated by the patient twice a day using a visual analogue scale (VAS) scoring 0–10 (0 no pain at all, 10 intolerable pain). The tolerability of the mouthwashes was evaluated by the pa- tient once daily using a VAS scoring 1–5 (1 very tolerable, 5 intolerable). The maximum body temperature during a day was recorded. Blood cultures from a peripheral vein and a central venous catheter were taken on the first epi- sode of fever (≥38.0°C) post-transplant and then whenever indicated by the medical staff. Oral cavity smears for mi- crobiological testing were carried out only in patient with clinical suspicion of a local infection.	Intervention was significantly less tolerable for patients ($P = 0.02$). No significant difference was found between both groups in respect of OM characteristics, fever of unknown origin and other infections. OM occurrence was significantly higher in females than males with increased severity and duration ($P = 0.0016$).
53	Dos Reis, P. E., Ciol, M. A., de Melo, N. S.,et al. (2016)	38 patient with gastric or colorectal cancer scheduled to receive chemotherapy, 18 years and above	Cryotherapy made with chamomile infusion at 2.5%	crytherapy with ice made of pure water.	Occurrence of OM, intensity of om measure by WHO assessment scale, mouth pain (1-10), presence of erythema, ulceration, mouth dryness, dietary category, presence of bleeding.	50% of the patients in the control and 30 % in the chamomile group developed oral mucositis. Mouth pain score was higher in patients in the control group on all evaluations (p= 0.02 for day 8, p

						= 0.09 for day 15, and p= 0.14 for day 22). Patients in the chamomile group never developed mucositis with grade 2 or higher. The occurrence of oral mucositis was lower in patients who used cryotherapy made with chamomile infusion than in patients who used cryotherapy made only with water. When compared to the control group, the chamomile group presented less mouth pain and had no ulcerations.
49	Gori, E., Arpinati, M., Bonifazi, F., et al. (2007).	122 patients undergoing myeloablative pretransplant conditioning, post transplant GVHD prophylaxis with MTX, age of at least 8 years old.	Cyrotherapy	Control	The primary end point of the study was the incidence of severe (grade 3–4) oral mucositis.	The incidence of severe (grade 3–4) oral mucositis, the primary end point of the study, was comparable in patients receiving or not cryotherapy. Moreover, no difference was observed in the incidence of oral mucositis grade 2–4 and the duration of oral mucositis grade 3–4 or 2–4. Thus, cryotherapy during MTX administration does not reduce severe oral mucositis in patients undergoing myeloablative allogeneic
50	Kamsvåg, T., Svanberg, A., Legert, K. G., et al. (2020).	**53 Swedish-speaking children between 4 and 17 years admitted for autologous or allogeneic HSCT.**	Oral cryotherapy	Control	Mucositis was measured using the WHO-Oral Toxicity Scale (WHO-OTS), scoring from 0 to 4. The child recorded symptoms of mucositis by using the Children's International Mucositis Evaluation Scale (ChIMES)	Severe OM (WHO-OTS ≥ 3) was recorded in 26 children (OC = 15, control = 11). OC did not reduce the incidence of severe OM, oral pain, or opioid use. No indication that OC reduced the incidence of OM in children undergoing HSCT in this study.
51	Karagözoğlu, S., & Filiz Ulusoy, M. (2005).	60 patients undergoing chemotherapy in respiratory diseases clinic of the Cumhuriyet University Faculty of Medicine during May 1999–May 2000.	Oral cryotherapy	**Control**	Patient-judged mucositis grades were used to measure the severity of mucositis. The investigator has used the inventory called as physician-judged mucositis grading in the literature after a preliminary study.	According to Patient-Judged Mucositis Grading, the rate of mucositis is 36.7% in study group and 90.0% in control group, the difference between two groups being statistically significant (P<0.05). According to Physician-Judged Mucositis Grading, the rate of mucositis is 10.0% in the study group and 50.0% in the

						control group, the difference between two groups being statistically significant(P<0.05).
52	Alkhouli, M., Laflouf, M., & Alhaddad, M. (2021).	26 children with acute lymphoblastic leukemia (ALL) aged 3-6 yo	70% aloe-vera solution	sodium bicarbonate 5%	World Health Organization grading scale	chemotherapy-induced oral mucositis degrees were less severe in the aloe-vera group than in the sodium bicarbonate group. Statistically significant difference of occurrence of different CIOM degrees between groups was recorded in the 2nd, 3rd, 4th, and 7th weeks of follow-up period. Moreover, Mann–Whitney U test indicated that patients in the sodium bicarbonate group (week 2.27) began CIOM sooner than those in the aloe-vera group (week 4.28) with a statistically significant difference (p = .001). Topical application of aloe-vera solution is effective in the prevention of chemotherapy-induced oral mucositis in ALL children.
59	Bahrololoomi, Z., Sadat-Hashemi, A., Hassan-Akhavan-Karbassi, M., et al. (2020).	44 patients (6-12 year old children) diagnosed with hematologic malignancies	Persica oral drops	normal saline	oral condition using a questionnaire consisting OAG index.	In the detailed analysis of the distribution of participants according to each of 8 factors included in OAG score (Table 2), none of them showed significant difference between treatment groups in either of sessions (P>0.05). Persica oral drop did not show significant additive effect on chlorhexidine for reducing chemotherapy induced mucositis of children in our sample size.
54	Elhadad, M. A., El-Negoumy, E., Taalab, M. R., et al. (2022)	45 patients, 30 or older, who are scheduled to receive 5-fluorouracil-based chemotherapy.	Test 1: chamomile topical gel 3% alone, 3 times/day for 3 weeks<br>Test 2: chamomile topical gel in combination with conventional symptomatic relief.	Conventional symptomatic treatment: antifungal agents, topical anaesthetics, anti-inflammatory agent 3 times/day, 1 day prior to chemotherapy, lasting 3 weeks.	OM severity asessed using WHO grading system, and pain using numeric rating scale (1-10) at week 1, 2 and 3.	Although OM severity during week 1, 2 and 3 did not show significant difference between the 3 groups, most patients experienced oral mucositis with more severity reported in the conventional group (grade III = 6.7%) compared to the other two groups, neither of which developed more than grade II.

						Mean pain scores showed no significant difference between the groups, but intragroup analysis showed that pain score increased in the conventional treatment group more than the other two groups.<br>Topical chamomile 3% gel has demonstrated in this study to lower the severity of the mucositis with lower pain scores compared to the other two groups.
58	Fidler, P., Loprinzi, C. L., O'Fallon, J. R., et al. (1996).	164 patients scheduled to receive their first course of a 5-FU-based, five consecutive day chemotherapy regimen	Chamomlie Mouthwash	Placebo mouthwash	Evaluate the prevention of 5-FU-induced oral mucositis using stomatitis scores given by attending physicians. Scores were given between 0 and 4 (0 being none and 4 being the most severe).	There was no substantial difference in stomatitis scores between the two protocol arms.
55	Hasheminasab, F. S., Hashemi, S. M., Dehghan, A., et al. (2020).	19 patients aged between 17 and 65 years, histopathologically confirmed breast cancer appropriate for treating with chemotherapy by a regimen consisting of Adriamycin, and having the reading and writing skills to fill the questionnaire	herbal preparation from Plantago ovata hydrocolloid	**Placebo**	The degree of mucositis was used as the main treatment outcome.	Compared with the placebo, the herbal compound significantly reduced the degree of mucositis, the severity of pain and the xerostomia grade; it also improved the patients' quality of life ($P < 0.05$).<br>Comparison between the screening cycle and placebo treatment group showed that the oral care protocol had a significant effect in the reduction of OM ($P < 0.05$).
56	Kobya Bulut, H., & Güdücü Tüfekci, F. (2016)	The 83 participants had received previous and new leukemia diagnoses or a lymphoma diagnosis and had also received treatment at the in-patient services and out-patient services departments, they were not diabetic or allergic to honey and received no honey from family members except during the study intervention, and they were between 6 and 17 years old. Children given methotrexate had standard methotrexate treatment.	honey was either applied before or after the onset of OM depending on the situation + routine mouth care. (39)	routine mouth care (37)	World Health Organization Mucositis Assessment Index was used to observe/assess oral mucosa, mucositis development, and mucositis recovery among the children at the time they were given CT or at the time of mucositis diagnosis. Scale measurements were as follows: 0 indicated (no mucositis), 1 slight degree of mucositis, 2 moderate degree of mucositis, 3–4 (severe mucositis).	There was a significant difference between the two groups in terms of OM development before the study began (honey group: 3.37 ± 2.78; control group: 2.12 ± 2.22) ($p<0.05$)<br><br>The rate of use of mouth care products in the honey group decreased on the 16th and 21st days but increased in the control group and the difference between the groups was significant ($p < 0.05$)

						OM degrees of the children in the honey group before OM occurred gradually decreased after the initiation of honey use on the first day, and that a significant difference existed between the follow-up days and OM degrees (p < 0.05).
57	Kooshyar, M. M., Mozafari, P. M., Amirchaghmaghi, M. et al. (2017)	23 (12 control, 11 intervention)<br><br>Twenty-three patients who underwent chemotherapy for blood malignancies in the Department of Heamatology Imam Reza Hospital, Mashhad, Iran	quercetin (a natural flavonoid) 2 times daily	placebo	The World Health Organization (WHO) oral toxicity scale was used to evaluate OM	The incidence of mucositis was lower in the quercetin group, but mucositis was more severe in the intervention group, which may be due to lower oral health status in the intervention group. small sample size, not statistically significant.
39	Dazzi, C., Cariello, A., Giovanis, P., et al. (2003)	90 patients affected by solid tumours and undergoing high-dose chemotherapy with autologous peripheral blood stem cell transplantation rescue, >14 years old.	Granulocyte-macrophage colony-stimulating factor (GM-CSF) mouthwashes	placebo	Severity of OM evaluated daily using National Cancer Institute Common Toxicity Criteria. Oral pain score evaluated daily.	There is no significant difference in the incidence of OM in both groups (p=0.16), as well as the occurrence of grade 3-4 stomatitis (chi square test, not significant (no p value stated)). No statistically significant difference for mean duration of stomatitis (t test, not significant).
40	Foncuberta, M. C., Cagnoni, P. J., Brandts, C. H., et al. (2001).	152 patients (18–65 years) with a proven diagnosis of breast cancer, lymphoma, or other solid tumor types and a Karnofsky performance status of greater than or equal to 50%.	Transforming growth factor (TGF)-3 mouthwash	Placebo mouthwash	Prevention of Chemotherapy-Induced Oral Mucositis	There was no advantage of TGF-β3 treatment regarding the incidence (TGF-β3 four times daily versus placebo [46% versus 47%]), onset, or duration of NCI-CTC grade 3 or 4 OM. TGF-3 was not effective in the prevention of CT-induced OM.
49	Hosseinjani, H., Hadjibabaie, M., Gholami, K., et al. (2017).	80 adult patients with non-Hodgkin's lymphoma(NHL), Hodgkin disease (HD) or multiple myeloma (MM),undergoing autologous HSCT, were enrolled in the study by the principal investigator. All patients had adequate cardiac, pulmonary, renal and hepatic function, as determined by the institutional protocol and were at least 18 years old.	Erythropoietin (EPO) mouthwash	Placebo mouthwash	OM incidence, severity and duration were evaluated as primary study outcomes using five-grade World HealthOrganization (WHO) oral toxicity scale	The incidence of OM (grades 1–4) in the EPO mouth-wash group and control group was significantly different (27.5% vs 77.5%,p<0.001). The differences between Mean daily WHO grades of OM were significant from the day 7 until day 13 after administration of HDC in study groups. There was also a significant decrease in the incidence of OM grades 2–4 in EPO mouthwash group compared

						with control group (p=0.003). EPO mouthwash because of a clinically meaningful effect could be introduced as an outstanding agent for prevention of OM.
42	Abramoff, M. M., Lopes, N. N., Lopes, L. A., et al. (2008).	13 (range 7–23 y) undergoing osteosarcoma treatment, and high-risk acute lymphoid leukemia (ALL) treatment.	prophylactic laser-irradiation	placebo laser irradiation	National Cancer Institute's Common Toxicity Criteria, version 2.0.57, visual analog scale, Granulocyte levels.	At the third evaluation, 73% of the patients in the prophylactic laser group did not have mucositis, and in the placebo group 27% had no mucositis, a difference that reached statistical significance (p 0.03). In group 2, 27% had no OM and did not require therapy. In group 3, the patients had marked pain relief (as assessed by a visual analogue scale), and a decrease in the severity of OM. most patient did not develop OM when prophylactically irradiated, and there was quick recovery and pain relief for those in the therapeutic laser group, even in those with significant granulocytopenia, thus enabling them to better tolerate their chemotherapy regimens. This demonstrates that LLLT has both a preventive and a therapeutic role in those prone to develop OM.
43	Arbabi-Kalati, F., Arbabi-Kalati, F., & Moridi, T. et al. (2013).	48 adult patients (≥18 yo) undergoing chemotherapy	lazer on	lazer off	World Health Organization (WHO) criteria (20); xerostomia was graded from 1 to 4. Pain was evaluated based on visual analog scale.	Statistically significant differences in mucositis intensity between the two groups (P<0.005) . There were statistically significant differences in xerostomia intensity between the two groups: xerostomia intensity in the laser on group was less than that in the laser off group (P<0.005). Patient pain intensity exhibited statistically significant differences between the laser on and laser off groups, indicating that pain in the laser group was less intense than that in the laser off group (P<0.05).

						Low-power laser might decrease the intensity of mucositis.
44	Cruz, L. B., Ribeiro, et al.(2007)	60 patients aged 3 to 18 years treated with chemotherapy or HSCT.	low energy laser therapy	no laser treatment	Oral mucositis severity scored using the Common Toxicity Criteria scale of National Cancer Institute	On day 8, of 20 patients (36%) who developed mucositis, 13 of them were from the laser group and 7 from the control group. On day 15, of 24 patients (41%) who developed mucositis, 13 of them were from the laser group and 11 from the control group. There was no significant difference between groups concerning the grades of mucositis on day 8 (P = 0.234) or on day 15 (P = 0.208). This study showed no evidence of benefit from the prophylactic use of low-energy laser in children and adolescents with cancer treated with chemotherapy when optimal dental and oral care was provided.
45	Schubert, M. M., Eduardo, F. P., Guthrie, K. A., et al. (2007).	70 patients (20-69yrs) at risk for developing OM including patients scheduled to receive myeloablaive conditioning regimens utilising chemotherapy with and without total body irradiation (cyclophosphamide/ 1,200 cGy TBI; cyclophosphamide–VP16/TBI or multiple drug regimens such as busulphan–cyclophosphamide; etc.) and GVHD prophylaxis (allogeneic transplant recipients), randomised into 3 treatment groups: 650 nm laser, 780 nm laser and placebo.	Direct laser treatment to the lower labial mucosa, right and left buccal mucosa, lateral and verntral surfaces of the tongue, and floor of mouth with energy densities of 2 J/cm^2	placebo - laser handpiece was not connected to the laser unit	Primary outcome was the severity and pain scores of oral mucositis	Found a reduction in OMI scores with difference between 650nm and placebo approaching statistical difference (P = 0.06). No significant difference in unadjusted model but after adjusting for total body irradiation (TBI), 650nm vs placebo had signficant difference (P = 0.03). Patients treated with 780nm laser had reduced average OMI scores but were as likely as placebo patients to suffer severe mucositis over the observation period although not significant (P = 0.09).
47	Silva, L. C., Sacono, N. T., Freire, M. D. C. M., et al. (2015).	39 patients; 20 in treatment, 19 in control<br><br>Patients above the age of 14 and were scheduled to receive the conditioning regimen with high-dose chemotherapy with or without total body irradiation.	Low-Level Laser Therapy at 660nm, 40mW, and 4 J/cm^2 with oral hygiene protocol	Standardised oral hygiene protocol for Haematopoietic Stem Cell Transplantation (HSCT)	The primary outcomes of this study were the occurrence and severity of Oral Mucositis and the Quality of Life of patients according to the Functional Assessment of Cancer Therapy-Bone Marrow Transplantation and Oral Health Impact Profile scores, when the laser and	LLLT patients presented with WHO grade 1 OM with no WHO grade 3 in the LLLT group whereas OM presentation was distributed over grade 1-3 in control group. LLLT did not influence the oral and general health-related QoL of patients undergoing HSCT (P = 0.074).

		Age 14-63			control groups were compared.<br><br>The secondary outcomes were the progression of the mucositis and QoL from admission to hospital discharge, the influence of mucositis on Quality of Life, and the relationship between Functional Assessment of Cancer Therapy-Bone Marrow Transplantation and Oral Health Impact Profile scores.	Clinical improvement was present but no improvement in QoL.
79	Awidi, A., Homsi, U., Kakail, R. I., et al. (2001).	32 patients	pilocarpine tablets	placebo tablets	Mucositis was scored using (1) Score A: a modification of the score described by JP Donnelly and colleagues, (2) Score B: World Health Organization (WHO) score method, (3) Score C: in which the most prominent sign or symptom was given a score	The differences in mucositis score, between the OP and placebo groups were highly significant when comparing score A P<0.001, score B P<0.001, score C P<0.001 and the presence or absence of mucositis P<0.005. OP is an effective and safe treatment in the prevention of oral mucositis in standard dose chemotherapy
78	Nottage, M., McLachlan, S. A., Brittain, M. A. et al (2003)	Patients approached for this trial were all those attending the gastrointestinal medical oncology clinics at the Princess Margaret Hospital and receiving chemotherapy with 5FU and leucovorin (LV) on a 5-day bolus schedule.<br><br>The study was designed to accrue 158 patients, 79 in each treatment arm.	10ml sulcrafate suspension/mouthwash for 2 mins then swallow. Mouthwash was used four times daily. Treatment commenced on the 1st day of therapy and continued daily for 15 days.	placebo mouthwash	The primary outcome measure was the sum of 15 daily scores of severity of mucositis, completed by each patient from day 1 of the chemotherapy cycle through to day 15. For each daily assessment, the patients were asked to describe their symptoms on a scale ranging from 0 (No discomfort) to 4 (Marked discomfort). the total score represents. both severity and duration of mucositis.	No statistically significant difference between the two treatment groups was found for any of the outcome variables analyzed.<br>(duration, severity and incidence)
77	Shenep, J. L., Kalwinsky, D. K., Hutson, P. R., et al. (1988).	48 children and adolescents receiving standardised intensive remission-induction chemotherapy; randomly separated into two groups of 24 (Sucralfate (Age 10.4 ± 2.2) and Placebo (Age 11.9 ± 2.4)). Patients were older than 1 year of age who were entered in a multiagent chemotherapy trial (ANLL-83) for previously untreated acute	Sulcralfate Suspension	Placebo - same appearance and taste	Primary outcome was to determine the effectiveness of sucralfate suspension on preventing development of mucosal damage and promote healing of ulcers<br><br>Secondary outcome was to determine measure gastrointestinal bleeding, weight	patients receiving sucralfate reported less oral pain than patients receving placebo (P = 0.06). More moderate and severe oral ulceration in placebo group (P = 0.12).<br><br>"Accordingly, we conclude that the administration of sucralfate suspension has limited, if any efficacy in the prevention and

		nonlymphocytic leukemia from 1983 to 1987.			loss and infectious complications of mucositis.	treatment of chemotherapy-induced mucositis."
36	Giles, F. J., Miller, C. B., Hurd, D. D., et al. (2003).	323 patients, 7 years of age or older and scheduled to receive a cytotoxic regimen	Iseganan solution	Placebo solution	Development of OM by study day 21. The assessments were carried out to determine (1) presence or absence of UOM defined as any ulceration or pseudomembrane formation in any of 8 protocol-specified sites within the oral cavity; (2) score on the NCI CTC stomatitis (5-point scale)	Among all 323 patients, analyzed according to randomization assignment, 43% and 33% of iseganan and placebo patients, respectively, did not develop UOM. The p-value was not significant.
48	Guimaraes, D. M., Ota, T. M. N., Da Silva, D. A. C., et al. (2021).	80 pediatric patients diagnosed ALL with a minimum age of 4 years and a maximum 12 years with sufficient cooperation to accept the treatment and able to perform the visual analogue scale (VAS) and evaluation periods undergoing chemotherapy with high doses of MTX.	Light-emitting diode therapy (LEDT) devices	low-level laser therapy (LLLT)	Oral mucositis was assessed in accordance to the World Health Organization (WHO) score.	The data lacks statistical differences between the groups for the whole period of analysis ($p > 0.05$, Mann-Whitney U test). These findings suggest that LEDT has similar effects to LLLT to avoid and treat oral mucositis.
81	Oshvandi, K., Vafaei, S. Y., Kamallan, S. R. et al (2021).	96 (48 control, 48 intervention) patients with a cancer diagnosis selected from one oncology clinic in the west of Iran.<br><br>45 patients in the zinc chloride group, and 25 patients in the placebo group completed this study.	Mouthwash for the zinc chloride group containing zinc chloride 0.2%, greasy mint, preservative	mouthwash for the control group were similar to the intervention group but lacked effective material	The severity of oral mucositis was measured by using the world health organization criteria for grading of oral mucositis.	The prevalence of grades of oral mucositis between groups was significant at the end of the first ($p < 0.046$), second ($p < 0.01$) and third ($p < 0.01$) weeks.<br><br>Comparison of weights showed no significant difference between the groups at the beginning of the study ($p > 0.05$), But at the end, there was significant difference between the zinc chloride group with placebo group ($p < 0.01$).
80	Peterson, D. E., Barker, N. P., Akhmadullina, L. I. et al (2009).	patients 18 years of age or older with colorectal cancer (stages I to IV) who were undergoing chemotherapy as the primary treatment modality. Patients who were experiencing moderate to severe (ie, WHO grade ≥ 2) OM4 during the first cycle of chemotherapy were eligible.<br><br>99 (33 high dose, 33 low dose, 33 placebo)	high-dose rhITF (10 mg/mL), low-dose rhITF (80 mg/mL),	placebo oral spray	The primary objective of the study was to assess the efficacy of the two doses of rhITF oral spray compared with placebo in reducing the risk for clinically significant oral mucositis (WHO grade ≥ 2) arising from the stomatotoxic effects of the anticancer therapies.<br><br>secondary objectives were to determine the safety and duration of OM assessed by an objective measure, the Oral	The proportion of OM incidence is generally higher in the control groups, however it is only significantly different in the OM-grade 2 comparison(9.5% & 48.5%; $P < .001$). there was a single case of OM-3 in treatment and no OM-4<br><br>There is a significant difference in the OMAS severity score between treatment groups and control ($p<0.01$)

					Mucositis Assessment Scale (OMAS)	
70	Piredda, M., Facchinetti, G., Biagioli, V. et al (2017).	Patients were eligible if they were ≥20 years old, were recently diagnosed with stage 0 to III breast cancer, had indication for chemotherapy with doxorubicin and cyclophosphamide (AC), had an Eastern Cooperative Oncology Group (ECOG) performance rating ≤2, platelet count ≥100 × 109/L, and Hgb > 9 g/dL.<br><br>60 (30 control, 30 intervention)	mouth rinsing with sodium bicarbonate three times a day plus tablets of a dry extract of propolis divided into 2–3 times/day between meals. Each tablet contained 80 mg of propolis titrated in galangin 8%–12%. The total daily number of tablets was calculated according to patient weight and ranged 8–10 mg/kg/day of propolis	mouth rinsing with sodium bicarbonate three times a day	The incidence and severity of OM, oral pain, use of opioids, compliance with the intervention, and adverse effects were evaluated in both groups after 5, 10, 15 and 21 days of the first chemotherapy cycle. OM was assessed using the National Cancer Institute Scale (NCI-CTCAE) version 4.0<br><br>Oral pain was assessed through a numeric pain rating scale (NPRS) ranging from 0 = no pain to 10 = the worst possible pain	No patient in the experimental arm developed oral mucositis graded higher than G1 during the first cycle. In the control arm, OM higher than G1 was developed during the first cycle in five (16.7%; n = 4 G2, n = 1 G3) patients (p = .02; chi-square test = 5.455; df = 1).<br><br>However the incidence of OM is not significant between the 2 groups.
82	Verdi, C. J., Garewal, H. S., Koenig, L. M., et al. (1995).	10 patients with a histologically documented carcinoma amenable to chemotherpy with bolus cisplatin and infusional 5-fluorouracil (5-FU). Age < 18 years, Karnofsky performance status <50%, prior oral radiation therapy, active and untreated oral infection, chemotherapy within 6 weeks of study entry, current use of pentoxifylline, creatinine > 1.5 mg/dl, white blood cell count < 4,000 mm^3, platelet count < 100,00/mm^3, concomitant administration of glucocorticoids, allopurinol, or ciprofloxacin, intolerance to xanthene derivatives, and life expectancy < 2 months.<br><br>Age range = 57-76	400mg Pentoxifylline 4x daily beginning 3 days before starting chemotherapy and continuing 7 days after treatment completion (15-day course)	placebo	Primary outcome was measuring the effectiveness of preventing CT-induced OM.<br><br>Oral assessment of oral mucositis included assessment of voice, swallow, lips, tongue, saliva, mucous membranes, gingiva and teeth to assess the function and integrity of the oral mucosa. Graded numerically with a score of one, two, or three<br><br>A score of one is normal function or integrity and a score of two or three represents mild or severe alteration of function or integrity respectively. The eight categoric scores are then added to yield a total score. A total score of eight is normal function and integrity with the high- est attainable score of 24 representing the most severely compromised patient.	No cytoprotective effect for pentoxifylline over placebo (P = 0.45)
83	Yüce, U., & Yurtsever, S. (2019).	60 patients above the age of 18 receiving chemotherapy for the first time, not receiving any education related to oral mucositis previously, having physical and cognitive health level. (30 intervention, 30 control).	Education about Oral Mucositis	No Education about Oral Mucositis	Primary Outcome - Oral Mucosa Assessment<br><br>Secondary outcome - patient's QoL and Pain	It was determined that there is a statistically significant difference between the education and control groups of degree of oral mucositis on the 5th day (p = 0.003), 10th day (p = 0.003), 15th

						day (p < 0.001), and 21st day (p < 0.001) of treatment<br><br>It was found to be a statistically significant difference be- tween education and control groups in terms of experiencing the following symptoms; "reduced saliva" on the 5th day of treatment (p = 0.005), "difficulty swallowing" on the 5th day of treatment (p = 0.018) and "deterioration in taste" on the 5th day of treatment (p = 0.004)
72	Arbabi-kalati, F., Arbabi-kalati, F., Deghatipour, M., et al. (2012).	50 adult patients (over 18 years of age) who underwent chemotherapy	three, 220 mg zinc sulfate capsules daily	three placebo capsules daily	World Health Organization (WHO), xerostomia, visual analog scale and questionnaire (EORTC LQ-OES18)	The results of the present study show that using zinc sulfate can signicantly decrease mucositis intensity and xerostomia (p<0.05) in patients who suffer from different malignancies and are under chemotherapy treatment.
61	Immonen, E., Aine, L., Nikkilä, A., et al. (2020).	56 Patients between the ages of 2-17.99 years who were diagnosed with a solid or hematological malignancy and who were receiving chemotherapeutic drugs. Their chemotherapy regimen included one of the following drugs that are known to expose to mucositis.	Caphosol mouthrinse	Saline mouthrinse	Oral changes and symptoms were evaluated using the World Health Organisation (WHO) toxicity scale and the Children's International Mucositis Evaluation Scale (ChIMES). The primary endpoint was the frequency and severity of OM and oral symptoms.	The peak of symptom scores was evident at around day 4-7 after administration of the chemotherapy with no marked differences between the rinse solutions. Multivariable regression analysis did not indicate a benefit of using Caphosol over the saline solution. No difference in prevention of oral mucositis was observed between the use of Caphosol or saline rinses.
71	Jahangard-Rafsanjani, Z., Gholami, K., Hadjibabaie, M., et al. (2013).	77 Adult patients with AML or ALL, undergoing allogenic HSCT. All patients had adequate cardiac, pulmonary, renal and hepatic function, as determined by the institutional protocol.	Selenium tablet	Placebo tablet	Grade of OM was the primary outcome.<br>It was evaluated with the use of five-grade World Health Organization<br>(WHO) oral toxicity scale	The incidence of severe OM (grades 3–4) was significantly lower in the selenium group (10.8% vs 35.1%, P<0.05). Selenium can reduce the duration and severity of OM after HDC.
69	Kitagawa, J., Kobayashi, R., Nagata, Y. et al (2021)	88 adults (control = 47, prevention = 41)<br><br>Patients with hematological malignancy undergoing HSCT were	polaprezinc lozenge	no intervention	the primary endpoint of our study was the incidence of Grade ≥3 oral mucositis. Secondary endpoints were the incidence and severity of oral mucositis, xerostomia and taste	PZ lozenges significantly reduced the incidence of Grade ≥2 oral mucositis (44.7% in control group vs 22.0% in the prevention group, P = .025),

64		enrolled between January 2017 and March 2019			disturbance. The incidence of other nonhematological AEs, the median time of engraftment and rate of engraftment were also compared between the two groups.	The lozenges did not prevent grade ≥3 mucositis.
64	Peterson, D. E., Jones, J. B., & Petit, R. G. (2007).	Adult patients (≥18 years of age) with histopathologically confirmed breast cancer suitable for treatment with anthracycline-based chemotherapy<br><br>326 (162 control, 163 intervention)	Saforis (glutamine) was administered at a dose of 2.5 g per 5 mL 3 times per day for a total daily dose of 7.5 g.<br><br>Study drug treatment began on the first day of chemotherapy and continued for 14 days after the last dose of chemotherapy in patients who did not develop OM or until 5 days after resolution of OM for patients who experienced OM or to the end of the treatment cycle. Study drug was orally swished for 30 seconds and then swallowed.	The placebo formulation matched the texture and characteristics of the active drug and the administered dose was 5 mL 3 times per day for a total daily dose of 15 mL.	The WHO OM scale and the OMAS were used to assess the severity of OM	During Treatment Cycle 1 the incidence of WHO grade ≥2 OM was significantly reduced for patients treated with Saforis compared with patients treated with placebo (38.7% vs 49.7%; P = .026)<br><br>The severity of OM in Treatment Cycle 1 showed a statistically significant (P = .042) drop in the Saforis arm<br><br>Although overall incidence of WHO grade ≥3 OM was low, the incidence was significantly lower in the Saforis arm compared with the placebo arm (P = .005).<br><br>Treatment with Saforis was also associated with a statistically significant decrease in worst ulceration score in Treatment Cycle 1 compared with patients receiving placebo (P = .013)
76	Mansouri, A., Hadjibabaie, M., Iravani, M. et al(2012).	Suitable patients (n=60) were all adults 15 years or older, with hematologic malignancies who were undergoing high-dose chemotherapy conditioning regimen for allogenic HSCT.	zinc sulfate capsule 220 mg (50 mg zinc elemental) twice daily	placebo	The primary outcome was the severity of mucositis after received the same prophylaxis regimen for prevention of mucositis, which included 20 drops of nystatin every 3 h, chewable tablet sucralfate 500 mg every 8 h, and mouth washes such as 10 cc chlorhexidine 0.02%, diluted vial of amphotericin, and 10 cc diluted povidone iodine every 3 h.	There was no significant difference in zinc serum concentrations between experimental group and placebo group (p-value = 0.178)<br><br>Mucositis developed in 25 patients in group A and 23 patients in group B, although the difference was not significant (p-value = 0.748).
75	Mehdipour, M., Taghavi Zenoz, A., Asvadi	30 patients were selected, and matched with respect to medical history, tumor characteristics, and	rinse with 10 ml of 0.2% zinc sulphate mouthwash two times par day for 14 days	10 ml of 0.2% chlorhexidine	The efficacy of the treatment at each session was determined by recording the length (E) of the	mean mucositis score was generally lower in the test group compared to the controls at all

	Kermani, I et al. (2011).	therapeutic details. Subjects were allocated to experimental and control groups of 15 patients each.		mouthwash in the same manner	lesion, measured by a digital caliper, and its severity (K), based on local signs, according to the Spijkervet scale	four time intervals evaluated, repeated measure ANOVA revealed that the difference was statistically significant in weeks 2 and 3 (P=0.025).
67	Moslehi, A., Taghizadeh-Ghehi, M., Gholami, K. et al(2014).	Adult patients with AML, ALL or myelodysplastic syndrome (MDS) were recruited into the study and received BU and CY before allogeneic hematopoietic SCT. Patients had normal cardiac, hepatic and renal functions and were at least 18 years old. Patients who had a Karnofsky performance status <70% were excluded from the study.<br><br>80 (42 control, 38 prevention)	100 mg/kg body weight injectable NAC N-acetyl cysteine (Exir Pharmaceuticals Company, Boroujerd, Iran, 2g/10 mL ampoules) that was diluted in 500 mL dextrose solution 5% and administered as an intravenous infusion over 3 h every day, from the starting day of HDC until day +15 after transplantation.	10 mL sterile water for injection ampoules	OM incidence, severity and duration were assessed as primary study outcomes using five-grade World Health Organization (WHO) oral toxicity scale.	The incidence of OM (grade 1–4) in the NAC group and control group was not significantly different (92.1% vs 97.6%, P=0.34)<br><br>The incidence of severe OM (grades 3 and 4) was significantly lower in the intervention group (P=0.04)<br><br>The mean (s.d.) duration of OM was significantly shorter among the NAC recipients (6.24(2.96) days vs 8.12(3.97) days, P=0.02). There was no significant difference in the time to onset of OM between two groups.
74	Rambod, M., Pasyar, N., & Ramzi, M. (2018).	all adult leukemia patients treated with chemotherapy<br><br>86 (34 control, 34 intervention)	Zinc sulfate capsule containing 50 mg zinc	placebo capsule	objective and subjective evaluation of mucositis, time of beginning of mucositis, and prevention of mucositis. The subjects were evaluated on 4th, 7th, and 14th days after chemotherapy<br><br>evaluation is done by a mucositis index and VAS	During the 14 days of the study, 27 subjects in the experimental group (75.00%) and 17 ones in the control group (47.22%) did not have the signs and symptoms of mucositis. The results of Chi-square test showed a significant difference between the experimental and control groups regarding the prevention of mucositis ($\chi 2 = 5.84$, $p = .01$).<br><br>Experimental group has significantly lower severity score, lower incidence rate. however, the onset of OM has no significant difference between the 2 groups.
65	Sornsuvit, C., Komindr, S., Chuncharunee, S., et al. (2008).	16 patients (Age 15-70) receiving chemotherapy for acute myeloid leukaemia	glutamine dipeptide supplementation on days 1-5 of chemotherapy	standard amino acid mixture	primary outcome was to measure the neutrophil phagocytic function<br><br>secondary outcome was to measure effectiveness of	higher phagocytosis levels and superoxide anion generation in glutamine supplement group vs control. however both groups had significantly lower values of phagocytosis levels than normal

					treatment based on severe oral mucositis (grades 3 and 4); severe diarrhoea (grades 3 and 4); prolonged diarrhoea (> 10 days); and prolonged neutropenia (> 14 days).	values<br><br>No significant difference (P > 0.05) between the glutamine supplement group and control groups in terms of total cost of hospital stay. Chances of being free from infection, severe diarrhoea or prolonged neutropenia were same in both groups, severe mucositis and prolonged diarrhoea were not statistically significantly less prevalent in the glutamine supplement group than in the control group.
62	Tanaka, Y., Takahashi, T., Yamaguchi, K., et al. (2016)	30 patients at least 18 years of age and have histologically or cytologically confirmed oesophageal squamous cell carcinoma or adenocarcinoma	Glutamine; Glutamine + Elemental Diet	No treatment	Primary outcome was to measure the efficacy and safety of Elental + Glutamine and Glutamine alone in comparison to no treatment<br><br>Secondary outcome was to measure and compare the mucosal integrity and degree of inflammation between treatment groups	Lower significant incidence of oral mucositis in Glutamine + Elemental Diet group compared to control during the first cycle of chemotherapy (P = 0.040). No significant difference was observed in the second cycle. Glutamine + Elemental Diet (P = 0.02) and Cancer stage (P = 0.01) were independent factors affecting mucositis grade during chemotherapy.<br><br>No significant differences in other parameters that reflect systemic inflammatory status and nutritional index. Lower plasma concentrations for some amino acids in control group.<br><br>Body weight was maintained in the Glutamine + Elemental Diet Group whereas control group lost weight -> significant difference in weight loss (P = 0.01).
63	Toyomasu, Y., Mochiki, E., Yanai, et al. (2019)	22 patients aged 20-80 (11 treatment, 11 control) that were undergoing chemotherapy for gastric cancer. The patients received 80	Elemental Diet (Elental)<br><br>The supplements were consumed as a solution of one pack with 250 mL water (total volume =	Control - No Elental	Primary outcome was the presence and grade of oral mucositis - grades of oral mucositis and diarrhoea were determined in accordance with	The incidence of oral mucositis was significantly lower in the treatment group (9.1%) than in the control group (27.3%) (no P-value)

		mg/m2 of S-1 on days 1–28 of every 42- day cycle	300 mL liquid) or with 150 mL water plus agar powder (total volume = 200 mL jelly). Patients could select either form of Elental® at any time of the day, regardless of meal times.		the Common Terminology Criteria for Adverse Events (CTCAE v4.0), Oral mucositis was assessed by independent physicians<br><br>Secondary outcome was adherence to Elental based on doses recorded in a diary, changes in nutritional parameters (serum total protein, serum albumin, retinol binding protein, transthyretin, and transferrin), and frequency and severity of adverse events.	Amino-acid rich elemental diet (Elental) may be useful as a countermeasure for S-1 adjuvant chemotherapy-associated mucositis. Continuation of S-1 adjuvant chemotherapy was higher than control. Lower incidence of oral mucositis in the treatment group vs control
66	Widjaja, N. A., Pratama, A., Prihaningtyas, R., et al. (2020).	48 children (Age 1-18) who underwent consolidation phase chemotherapy and received high-dose methotrexate; undergoing chemotherapy for acute lymphoblastic leukemia (24 intervention, 24 placebo)	oral glutamine (400mg/kg body weight per day) received on days of chemotherapy treatment until completion of therapy glutamine and placebo were given for 14 days	placebo	Primary outcome was to evaluate the incidence of OM, duration of treatment and cost of care.	Oral mucositis occurred in 4.2 % of the glutamine group and 62.5% in the placebo group. The use of glutamine was directly associated with prevention of oral mucositis than placebo (OR 0,026; 95% CI: 0,003-0,228). Significant difference in the incidence of OM between intervention and control (P < 0.05). The duration of length hospital stay was lower in the glutamine group than in the placebo group ((8 vs 12 days); p = 0,005).
73	Gholizadeh, N., Mehdipour, M., Chavoshi, S. H., et al. (2017).	140 Patients, 18 -71 years of age, undergoing chemotherapy with AML, and chemotherapy treatment by a regimen with the same mucositis probability.	Zinc sulfate capsules	Placebo capsules	Oral mucositis was graded from 0 to 4, using World Health Organization (WHO) criteria.	The frequency of severe mucositis (grade 3 - 4) was significantly lower in case group (1.4%) than in control group (7.2%) at the end of the 4th week of treatment (P = 0.004).
60	Hamidieh, A. A., Sherafatmand, M., Mansouri, A., et al. (2016).	28 Patients with Fanconi anemia aged 1–15 years, undergoing allogeneic HSCT	Calcitrol	Placebo capsule	Severity, time of onset, and recovery of OM was assessed using the World Health Organization oral toxicity scale.	In this study, calcitriol did not change OM incidence and severity significantly. In conclusion, we did not find considerable benefits of calcitriol in the prevention of OM.
68	Hashemipour, M. A., Barzegari, S., Kakoie, S., & Aghahi, R. H. (2017).	60 patients with malignant tumors undergoing chemotherapy treatment for the first time developing World Health Organization (WHO) grade 1 oral mucositis	Omega-3	Placebo	Mucositis was assessed according to the WHO, Western Consortium for Cancer Nursing Research, and Oral Mucositis Weekly Questionnaire criteria	At 1 and 2 weeks after starting the study, the severity of mucositis was significantly lower in the omega-3 group compared with the control group. After 2

						weeks, there was no evidence of mucositis in the patients taking omega-3. The difference between the 2 groups was significant (P = .002).
111	Rosen, L.S., Abdi, E., Davis, I.D., et al (2006).	64 patients with metastatic colorectal cancer (CRC) receiving fluorouracil/leucovorin (FU/LV) chemotherapy.	palifermin (40 microg/kg for 3 consecutive days)	Placebo	incidence of OM and diarrhea, safety, disease progression, and survival	The incidence of WHO grade 2 or higher OM was lower in patients who received palifermin compared with placebo (29% v 61% in cycle 1; 11% v 47% in cycle 2). FU dose reductions in the second chemotherapy cycle were more frequent in the placebo group (31%) than in the palifermin group (14%).

Table S3. Summary finding of article reporting preventative interventions for radiochemotherapy-induced OM

Ref. NO	Author, Year	Patient Population and Size	Intervention	Control/Comparison	Outcomes	Summary of Findings
85	Chitapanarux, I., Tungkasamit, T., Petsuksiri, et al. (2018)	60 non-metastatic head and neck cancer patients, aged 18-70 yrs old, scheduled to be undergoing platinum-based concurrent chemo-radiotherapy (CCRT) at least 60 GY.	0.15% benzydamine hydrochloride mouthwash	0.15% sodium bicarbonate mouth wash	Severity of OM measured weekly using Oral mucositis assessment scale (OMAS). Pain score (0-10) and oral candidiasis assessment were assessed.	The median of total OMAS score was statistically significant lower in patients who received benzydamine HCl during concurrent chemo-radiotherapy (CCRT) than in those who received sodium bicarbonate, (p value < 0.001). There was no difference in median pain score, (p value = 0.52). Prophylaxis oral rinsing with benzydamine HCl for patients undergoing high-dose radio-chemotherapy was superior to sodium bicarbonate mouthwash in terms of alleviating the severity of oral mucositis.
9	Rastogi, M., Khurana, R., Revannasiddaiah, S. et al (2017).	120 patients (60 control, 60 intervention) of histopathologically proven squamous cell carcinoma of the head and neck with age >18 years, Karnofsky performance status (KPS) ≥70, planned radiation dose of ≥60 Gy (either definitive or post-operative), hemoglobin ≥10 g/dL, leukocyte count ≥4000/mm3, absolute neutrophil count ≥1500/mm3, platelets ≥100,000/mm3, creatinine clearance ≥50 mL/min, and normal liver function tests	saline mouth rinses + benzydamine rinses (0.15%)	saline mouth rinses	Patients were examined weekly during and till 4 weeks after completion of RT. Mucositis and pain were recorded and graded as recommended by WHO and CTCAE (common terminology criteria for adverse events version 4.0)	Patients in experimental radiotherpay group had lesser grade 3 WHO-M and CTC-M as compared to control radiotherapy group, 62.1 vs. 36.4% (p = 0.038) and 51.7 vs. 27.3% (p = 0.043), respectively<br><br>There is no significant difference in WHO-M and CTC-M in the experimental and control chemotherapy groups.<br><br>Benzydamine oral rinses in addition with saline rinses significantly reduces the rates of oral mucositis in patients of head and neck cancers treated with radiation doses >50 Gy and up till 70 Gy. (p<0.05)
84	Diaz-Sanchez, R. M., Pachón-Ibáñez, J., Marín-Conde, F., et al. (2015)	7 patients aged 18- 65, having histological documented diagnosis of squamous carcinoma on the head and neck region in stage III and IV, and receiving combined radiotherapy and chemotherapy	topical application with chlorhexidine gel	placebo gel	Severity of OM using WHO criteria, pain score using VAS scale, adjuvant analgesics patient took, tolerance based on frequency of adverse event happened for the patient during the trial.	There is no signifiant difference between mucositis grade, pain and tolerance between the study group and the control group (p>0.05).

92	Arantes, D. A. C., da Silva, A. C. G., Freitas, N. M. A., et al. (2021).	62 patients received the following standard fractionation RT (intensity-modulated radiation treatment): 2.0 Gy/fraction, 5 days/week, during 6- or 7-week duration (35 sessions), and a total dose of ≥60 Gy.	FITOPROT (mucoadhesive formulation containing 20 mg/ml curcuminoids [>95% purity, C7727, SIGMA] and 40% glycerinated Bd extract)	mucoadhesive formulation without CL and Bd extracts	WHO and NCI scales	FITOPROT prevented the occurrence of OM that was considered to be ≥grade 3 in 83.3% (n = 15) of the patients with cancer allocated to the intervention group, while the placebo prevented it in 53.3% (n = 7) of the patients with cancer when the entire period of clinical evaluation was considered (Pearson's chi-squared, p > 0.05, WHO and NCI). There is evidence that FITOPROT in mouthwash form does not promote adverse effects in patients with cancer and that it presents clinical effectiveness in reducing the severity of OM and biological effectiveness in controlling inflammation when compared to the placebo.
90	Babaee, N., Moslemi, D., Khalilpour, M., et al. (2013).	40 patients with a diagnosis of head and neck cancers	2% calendula extract mouthwash	placebo mouthwash	OM severity in all patients was measured using Oral Mucositis Assessment Scale (OMAS) scores.	According to repeated measures ANOVA test, the differences between OMAS of calendula and placebo during the weeks of evaluation were statistical significant (p < 0.001) and OM intensity was significantly decreased in calendula compared to placebo group (p = 0.048). Calendula officinalis is effective on decreasing OM intensity but cannot completely prevent its occurance. All patients could tolerate it without any significant side effects (i.e., nausea and vomiting).
91	Kia, S. J., Basirat, M., Saedi, H. S., & Arab, S. A. (2021).	50 patients undergoing head and neck chemotherapy	Nanomicelle Curcumin capsules	placebo capsules	The WHO Mucositis Scale was used to assess the OM severity.	Oral mucositis severity in control group in the first (P = 0.010), fourth (P = 0.022) and seventh (P < 0.001) weeks were significantly more than the study group. Nabomicelle Curcumin capsules is effective on prevention and treatment of head and neck radiotherapy and especially chemotherapy induced OM.
89	Dantas, J. B. D. L., Martins, et al.(2020)	54 patients with malignant neoplasms in the head and neck region and aged 18 years or above.	low power laser device	placebo	Degree of OM assessed using WHO 1979 scouting scale of mucositis. Pain score measured using VAS (0-11).	There was no statistically significant difference between the laser and control groups regarding the development of OM (p>0.05) or pain perception (p>0.05).
87	Gautam, A. P., Fernandes, D. J.,	121 patients newly diagnosed with primary OC of squamous cell origin, 18 years or	He-Ne Laser	Placebo (sham laser therapy)	OM assessed using the Radiation Therapy Oncology Group/European Organization	Incidence of severe OM (29% vs. 89%, p < 0.001) was significantly less in laser than placebo group patients.

	Vidyasagar, M. S., et al. (2012).	older, scheduled to undergo chemoradiotherapy for primary oral cavity cancers.			for Re-search and Treatment of Cancer (RTOG/EORTC)22 scoring system.	
86	Khouri, V. Y., Stracieri, A. B., Rodrigues, M. C., et al. (2009).	22 patients subjected to allogeneic HSCT. 12 years of age or older, both genders, hematologic or onco-hematologic disease, myeloablative conditioning regimen, and allogeneic hematopoietic stem cell transplantation.	therapeutic laser	Conventional care (mouthwash called "Mucositis Formula")	Two scales were used for evaluation of OM: World Health Organization (WHO) Oral Toxicity Scale and Oral Mucositis Assessment Scale (OMAS).	Comparison of the course of OM between patients of groups I and II, showed that group I presented a lower frequency of OM and the difference between groups was statistically significant (p=0.02). Laser reduced the frequency and severity of OM, suggesting that therapeutic laser can be used both as a new form of prevention and treatment of OM.
46	Silva, G. B., Mendonça, E. F., Bariani, C., et al. (2011).	42 patients who underwent autologous or allogenic Haematopoietic Stem Cell Transplantation (HSCT) after chemotherapy with/without total body irradiation. These patients were scheduled to receive myeloablative conditioning regimens utilizing chemotherapy with and without total body irradiation. Median Age: Laser = 20.9; Control = 28.7.	Low-Level Laser Therapy at 660nm, 40mW, and 4 J/cm^2 with oral hygiene protocol	Oral Hygiene Protocol provided by Araujo Jorge Cancer Hospital/Goias Fight Cancer Association-Brazil	Primary outcome was to investigate the clinical effects of LLLT on the prevention of conditioning-induced OM in HSCT.	Laser application reduced the occurrence and intensity of oral mucositis and had a significant difference between intervention and control (P < 0.001). No patients in intervetion group developed grade 3 and grade 4 oral mucositis. Low-level laser therapy may be effective in preventing cancer-therapy induced oral mucositis in patients undergoing haemopoietic stem cell therapy.
88	Zanin, T., Zanin, F., Carvalhosa, A. A., et al. (2010).	72 patients (34-80yrs) with head and neck cancer, intact OM and were not receving drugs for the treatment or prevention of mucositis (36 intervention; 36 control)	Diode Laser λ = 660 nm, power = 30 mW, spot size = 2 mm, continuous mode. Energy used was at 2 J per point or scattering on the affected area with 1 cm^2 per application point.		Evaluate quantitatively and qualtatively the effect of laser in prevention and treatment of OM<br><br>Data were collected through daily physical intraoral ex-amination, and evaluation of OM was made according to the association of three criteria: National Cancer Institute (NCI) criteria, Brown scale to evaluate the incidence of OM, and the Visual Analog Pain Scale (VAS).	The results of evaluation according to the NCI scale can be found in Figure 1. Comparing the two groups, statistically significant differences were observed from the first week on (p < 0.001). Patients in group L did not present differences in the oral aspect during the experiment (p = 0.41) with an in-crease in the NCI scale results being observed during week 4 (p = 0.01) and returning to normal the following week. All patients in group C had OM levels varying from I to III on the NCI scale. This difference was significant from week 1 on (p < 0.001), increased until week 4 and then remained stable up to week 7 of the cancer treatment (p = 0.68).<br><br>Differences in OM results between the two groups was also significant when the Brown scale was used (p < 0.001).

						The absence of significant differences with the Brown scale results found in group L during the experimental period (p = 0.42) suggests that laser therapy was successful in preventing and controlling OM lesions. In the control group, OM incidence increased from week 1 until week 4 (p ≤ 0.001), remaining stable up to week 7 (p = 0.17), with moderate to severe OM lesions being observed (Fig. 2).
97	Chaitanya, B., Pai, K. M., Yathiraj, P. H., et al. (2017).	60 Patients diagnosed with Head & Neck cancer recruited for concurrent chemo-radiotherapy	Rebamipide gargle	Placebo gargle	assessment of oral mucositis was done by Numeric Rating Scale (NRS) and objective scoring according to RTOG system.	There was a delay of 3.5 days in the onset of oral mucositis in the Rebamipide group as compared to the Placebo group. (p = 0.012). At the end of radiation therapy, RTOG score of Group 1 subjects ranged from 1 to 3 with a mean score of 1.97 ± 0.61 whereas it ranged from 1 to 4 in Group 2 subjects with a mean score of 2.81 ± 1.02. (p = 0.001). Subjects of Group 1 also showed lower intensity of pain at the end of the treatment as compared to the subjects of Group 2. (p = 0.001). Rebamipide gargle is effective in delaying the onset of oral mucositis and also reducing the severity and pain intensity of oral mucositis in patients undergoing definitive chemo radiation for head and neck cancer.
99	Kawashita, Y., Koyama, Y., Kurita, H., et al. (2019).	124 patients with oral carcinoma scheduled to undergo radiation therapy with or without chemotherapy between September 2013 and December 2016.	Comprehensive oral management protocol (spacers to cover the entire dentition, pilocarpine hydrochloride, and topical dexamethasone ointment for oral mucositis)	Control (only oral care)	The primary outcome was the incidence of severe oral mucositis classified as grade 3 or higher according to the National Cancer Institute Common Terminology Criteria for Adverse Events version 4.0	The intervention was significantly associated with a decreased incidence of severe oral mucositis in patients receiving radiotherapy alone (P = 0.046), but not in those receiving chemoradiotherapy (P = 0.815). These findings suggest that an oral management protocol can prevent severe oral mucositis in patients with oral cancer undergoing radiotherapy without concurrent chemotherapy
98	Wu, S. X., Cui, T. T., Zhao, C., et al. (2010)	156 patients (53 prevention; 51 treatment; 52 control)	Actovegin Intravenous Actovegin of 30 ml daily (5 days/week)	No intervention	Evaluate efficacy of Actovegin in OM prevention and treatment	The incidence of grade 3 mucositis was lower in Group 1 (Actovegin before OM) compared with Group 3 (Control)

		patients with newly diagnosed stage III–IVB (UICC 2002 staging criteria) NPC scheduled for CRT, age 18–70 years, a Karnofsky performance score P 70, no significant oral disease, and normal cardiac, hepatic, renal, and hematopoietic functions.	was administrated from day 1 of the radiotherapy for Group 1 and from the onset of grade 2 mucositis for Group 2, until the end of the radiotherapy.		Acute toxicity of oral mucosa and oral pain were carefully ob- served and evaluated daily during the treatment according to NCI Common Toxicity Criteria (NCI CTC) 2.0 criteria and verbal rating scales (VRS) criteria [14], respectively.<br><br>The grading criteria of NCI CTC 2.0 were as follows: 0 = normal, 1 = erythema, 2 = patchy pseudomembrane, 3 = confluent pseudomembrane, and 4 = necrosis or deep ulceration.<br><br>The grading criteria of VRS were as follows: 0 = no pain, 1 = mild pain, sleep unaffected, 2 = moderate pain, sleep affected, and 3 = severe pain, sleep severely affected.<br><br>The time to the occurrence of grade 3 mucositis was recorded from day 1 of radiotherapy.<br><br>The primary endpoints were the incidence and the time of occurrence of grade 3 mucositis during concomitant chemoradiotherapy.	(P = 0.002). Group 2 (Actovegin at onset of OM) had a lower progression rate of mucositis from grade 2 to 3 compared with Group 3 (P = 0.035). There was no difference in the onset time of grade 3 mucositis among 3 groups.<br>Actovegin was well tolerated and no treatment-related adverse events were observed.<br>Actovegin is effective in the prevention and treatment of chemoradiotherapy-induced oral mucositis.
93	Cerchietti, L. C., Navigante, A. H., Lutteral, M. A., et al. (2006).	29 patients with head-and-neck cancer were treated with chemoradiotherapy	intravenous l-alanyl-l-glutamine 0.4 g/kg weight/day	equal volume of saline (placebo)	Mucositis was assessed by the Objective Mucositis Score (OMS) and the World Health Organization (WHO) grading system. pain. need for feeding tubes	There was a significant difference in incidence of mucositis developed in patients receiving placebo compared with those who received l-alanyl-l-glutamine (p = 0.035). The number of patients with severe objective mucositis (OMS >1.49) was higher in the placebo group compared with the l-alanyl-l-glutamine group (67% vs. 14%, p = 0.007). l-alanyl-l-glutamine patients experienced less pain (p = 0.008) and

						need for feeding tubes (14% vs. 60% respectively, p = 0.020) compared with placebo patients. Patients with head-and-neck cancer receiving CRT with a high yield of oral mucositis, intravenous l-alanyl-l-glutamine may be an effective preventive measure to decrease the severity of damage.
94	Chattopadhyay, S., Saha, A., Azam, M., et al. (2014).	70 biopsy proven patients with head and neck cancer receiving primary or adjuvant radiation therapy	oral glutamine suspension	nothing	WHO grading of mucositis, onset and duration of mucositis	Oral glutamine delays the development of mucositis. The mean time of onset of mucositis is significantly delayed in patients who received glutamine with P < 0.001. The mean duration of grade 3 mucositis or worse (grade 3 and grade 4) was significantly less (6.6 days vs. 9.2 days) in the glutamine arm.<br>The mean total duration of mucositis as well as all the different grades were significantly lesser in the glutamine arm. Although the incidence of mucositis as a whole was not decreased significantly, but incidence and duration of grade 3 and grade 4 mucositis were significantly less with glutamine with P values 0.02 and 0.04, respectively.
95	Dechaphunkul, T., Arundon, T., Raungkhajon, P., et al.(2022)	110 patients with HNC undergoing definitive CCRT including 3-week cycles of cisplatin, 18-65 years	Immunonutrition: omega-3-fatty acids, arginine, dietary nucleotides, and soluble fiber	placebo	The proportion of patients with grade 3-4 OM during CCRT between the 2 groups according to NCI CTCAE version 4.03.	There was no significant difference of the proportion of patients with grade 3-4 oral mucositis between the two groups (62% vs. 67%, p =0.690)<br>Not enough evidence to demonstrate immunonutrition reduced the risk of severe oral mucositis.
96	Watanabe, T., Ishihara, M., Matsuura, K., et al. (2010).	31 patients (age 35-86) with head and neck cancer who underwent RT or CRT; 16 intervention, 15 control	Polaprezinc (zinc L-carnosine)	azulene oral rinse	Primary endpoint was the prevention of oral mucositis<br><br>secondary endpoints included the prophylaxis of pain, xerostomia and taste disturbance, reduction in the use of analge- sics for the relief of oral pain, lowering the number of patients who showed inability to oral intake, and the preven- tion from reduction in the amount of daily meals.	The incidence rates of mucositis, pain, xerostomia and taste disturbance were all markedly lower in polaprezinc group than in control. Moreover, the use of analgesics was significantly (p = 0.003) less frequent and the amount of food intake was significantly (p = 0.002) higher in polaprezinc group than in control. On the other hand, tumor response rate in patients with neoadjuvant radiochemotherapy was not significantly affected by polaprezinc, in which the response rate (complete plus partial response) was

					The incidence of mucositis, pain, xerostomia and taste disturbance were evaluated and the severity was graded according to the Common Terminology Criteria for Adverse Events v3.0 (CTCAE).	88% for polaprezinc and 92% for control (p = 1.000). Therefore, it is highly assumable that polaprezinc is potentially useful for prevention of oral mucositis and improvement of quality of life without reducing the tumor response.
110	Spielberger R, Stiff P, Bensinger W, et al. (2004).	double-blind study compared the effect of palifermin with that of a placebo on the development of oral mucositis in 212 patients with hematologic cancers	palifermin (60 microg per kilogram of body weight per day)	Placebo (i.v.)	Incidence and severity of oral mucositis; edverse events	palifermin was associated with significant reductions in the incidence of grade 4 oral mucositis (20 percent vs. 62 percent, P<0.001), patient-reported soreness of the mouth and throat (area-under-the-curve score, 29.0 [range, 0 to 98] vs. 46.8 [range, 0 to 110]; P<0.001), the use of opioid analgesics (median, 212 mg of morphine equivalents [range, 0 to 9418] vs. 535 mg of morphine equivalents [range, 0 to 9418], P<0.001), and the incidence of use of total parenteral nutrition (31 percent vs. 55 percent, P<0.001).
112	Henke M, Alfonsi M, Foa P, et al (2011)	Multicenter, double-blind, randomized, placebo-controlled trial in 186 patients with stages II to IVB carcinoma of the oral cavity, oropharynx, hypopharynx, or larynx treated with postoperative radiotherapy plus cisplatin	palifermin 120 µg/kg from 3 days before and continuing throughout radiochemotherapy	Placebo	The primary end point was the incidence of severe oral mucositis (WHO grades 3 to 4). Overall survival and time to locoregional progression were also assessed.	Severe oral mucositis was seen in 47 (51%) of 92 patients administered palifermin and 63 (67%) of 94 administered placebo (P = .027). Palifermin decreased the duration (median, 4.5 v 22.0 days) and prolonged the time to develop (median, 45 v 32 days) severe mucositis.
